# Macrophage and mitochondrion dual-targeting astaxanthin nanoparticles prepared by Maillard reaction for colonic inflammation alleviation

**DOI:** 10.1007/s42995-024-00255-9

**Published:** 2025-02-17

**Authors:** Kangjing Liu, Xueying Tian, Siyuan Fei, Yukun Song, A. M. Abd El-Aty, Mingqian Tan

**Affiliations:** 1 State Key Laboratory of Marine Food Processing and Safety Control, Dalian Polytechnic University, Dalian, 116034 China; 2 School of Food Science and Technology, Academy of Food Interdisciplinary Science, Dalian Polytechnic University, Dalian, 116034 China; 3 National Engineering Research Center of Seafood, Dalian Polytechnic University, Dalian, 116034 China; 4 Collaborative Innovation Center of Seafood Deep Processing, Dalian Polytechnic University, Dalian, 116034 China; 5 Department of Pharmacology, Faculty of Veterinary Medicine, Cairo University, Giza, 12211 Egypt; 6 Department of Medical Pharmacology, Medical Faculty, Ataturk University, 25240 Erzurum, Turkey

**Keywords:** Astaxanthin, Whey protein isolate-mannose conjugates, Nanocarriers, Mitochondria targeting, Colon inflammation

## Abstract

This study demonstrated the design of whey protein isolate (WPI)-mannose (Man) conjugates with triphenylphosphonium bromide (TPP) through self-assembly to prepare macrophage and mitochondrion dual-targeting astaxanthin (AXT) nanoparticles (AXT@TPP-WPI-Man). The nanoparticles displayed spherical structures with a well-dispersed size of approximately 206.1 ± 39.2 nm, with good biocompatibility, stability, and targeting capabilities. In vitro experiments demonstrated the specific accumulation of AXT@TPP-WPI-Man in mitochondria and exhibited good targeting ability toward macrophages. The AXT@TPP-WPI-Man effectively reduced reactive oxygen species and preserved the normal mitochondrial membrane potential. The AXT@TPP-WPI-Man treated ulcerative colitis mice exhibited a 52.32% increase in colon length with significant improvement in weight loss, disease activity index scores, and reduced release of inflammatory cytokines. Immunofluorescence staining indicated AXT@TPP-WPI-Man alleviated ulcerative colitis by reducing M1 polarization in colonic macrophages while promoting M2 polarization. The dual-targeting AXT@TPP-WPI-Man has the potential to improve astaxanthin bioavailability, presenting a promising delivery method for the treatment of ulcerative colitis.

**Supplementary Information:**

The online version contains supplementary material available at 10.1007/s42995-024-00255-9.

## Introduction

Ulcerative colitis, an idiopathic chronic inflammatory disease, is one of the most prevalent inflammatory intestinal diseases and is on the rise rapidly worldwide (Kaplan and Ng 2016). Current available therapeutic approaches for ulcerative colitis treatments are mainly based on anti-inflammatory or immunosuppressive drugs, as well as biologic therapeutic agents (Xiao et al. 2013). However, some of these treatments have been restricted due to problems with long-term therapeutic efficacy and serious side effects. Astaxanthin (AXT), a xanthophyll carotenoid, is mainly found in some marine organisms, such as salmon, krill, and algae (Zhao et al. 2019). The presence of carbon‒carbon double bonds and β-ionone rings in astaxanthin endows astaxanthin with excellent antioxidant activities (Fakhri et al. 2018). Astaxanthin has received more attention because several animal and clinical studies confirmed it has anti-inflammatory (Park et al. 2018), antidiabetic (Takemoto et al. 2015), antitumor (Zhang and Wang 2015), and immunomodulatory (Sifi et al. 2016) functions. However, the poor water solubility, chemical instability, and low bioavailability of astaxanthin has constrained its application in the food industry. Moreover, these same characteristics also extremely limit its effective delivery and absorption in organs (Ambati et al. 2014).

To enhance the oral bioavailability of astaxanthin, delivery systems, including nanoparticles (Hu et al. 2020), emulsions (Shu et al. 2018), liposomes (Pan et al. 2018), and others, have been developed. Many food-grade proteins possess amphiphilic properties and have emerged as promising carriers for delivery due to their technological functionality and nutritional value. Whey protein isolate (WPI), abundant in essential amino acids, is an amphiphilic molecule with high surface activity and emulsifying properties (Shen et al. 2018). However, proteins are sensitive to factors, such as ionic strength, digestive enzymes, pH values, and high temperature, which can result in precipitation, aggregation, hydrolysis, and denaturation (Fan et al. 2018). Glycosylation of WPI offers a simple and effective approach to enhance emulsifying properties and antioxidant capacity. For instance, Jia et al. (2019) employed WPI-xylo-oligosaccharide conjugates prepared by the Maillard reaction to encapsulate lycopene, significantly improving its bioavailability. Similarly, Liu et al. (2017) demonstrated that glycosylation of whey protein conjugate microcapsules exhibited superior protection for probiotics under simulated gastrointestinal conditions.

Mannose (Man), a natural bioactive monosaccharide known for its anti-inflammatory and antioxidant properties, plays an important role in the signaling pathways of functional proteins on cell membranes (Zhang et al. 2017). Macrophages, which possess mannose receptors on their outer layer, present an attractive target for efficient delivery through mannosylated formulations (Martinez-Pomares 2012). Rifabutin-loaded mannosylated solid lipid particles have been developed and demonstrated improved uptake by macrophages in vitro (Nimje et al. 2009). Similarly, mannosylated liposomes have been evaluated for pulmonary administration of ciprofloxacin, showing significantly enhanced targeting efficiency toward alveolar macrophages as compared to unmodified liposomes (Chono et al. 2008). Naturally, glycosylated WPI-Man conjugates can serve as promising candidates for encapsulating astaxanthin due to their favorable biocompatibility and targeting capabilities. Mitochondria, the energy-generating powerhouses of cells, play a critical role in cell survival and death. Achieving highly selective mitochondrial targeting through nanotechnology can significantly enhance the bioavailability of astaxanthin. Triphenylphosphonium (TPP) bromide, a lipophilic cation with delocalized properties, has been utilized as a targeting moiety for mitochondria-specific delivery, as it readily accumulates in the mitochondrial matrix (Xu et al. 2017). Incorporating TPP into delivery carriers greatly facilitates their targeting of cellular mitochondria, thereby enhancing the efficiency of antioxidant activity (Jiang and Zhu 2019). By leveraging nanotechnology, the development of nanodelivery systems that effectively target macrophage mitochondria holds great potential for improving treatment effectiveness (Yu et al. 2023).

This study aims to develop dual-targeting nanocarriers that effectively deliver astaxanthin to alleviate inflammatory lesions in ulcerative colitis. The research objectives encompassed the following aspects: (1) the modification of triphenylphosphonium (TPP) bromide on WPI-Man conjugates, (2) the evaluation of loading capacity, stability, and mitochondrial targeting of AXT-loaded nanoparticles, and (3) the assessment of therapeutic effects through oral administration of the nanoparticles in dextran sulfate sodium (DSS)-induced colitis mice. Key parameters considered included disease activity index scores, colon length measurements, and histologic analysis. The findings of this study provide valuable insights into enhancing the bioavailability of astaxanthin and the potential application of these nanoparticle-based delivery carriers in the food industry.

## Materials and methods

### Materials

Astaxanthin (≥ 95%) was supplied by Aladdin Co., Ltd. (Shanghai, China). WPI was provided by Shanghai Yuanye Biotechnology Co., Ltd. (Shanghai, China). Mannose was procured from Sigma-Aldrich Co., Ltd. (St. Louis, MO, USA). TPP was obtained from Aladdin Co., Ltd. (Shanghai, China). DSS (MW: 36,000 ~ 50,000) was procured from Shanghai Advantage Biological Co., Ltd. (Shanghai, China). From the Nanjing Jiancheng Bioengineering Institute (Nanjing, China), a kit for measuring reactive oxygen species (ROS) was purchased. Beyotime Institute of Biotechnology Co., Ltd. (Haimen, China) provided the JC-1 assay kit for measuring mitochondrial membrane potential. Other reagents were of the analytical variety.

### Preparation of TPP-WPI-Man conjugates

The WPI-Man conjugates were made by thoroughly dissolving 4 g of WPI and 1% (w/v) mannose in 50 mL of deionized water. The solution was then lyophilized. Maillard conjugation reactions were conducted by placing the WPI and mannose in desiccators at 50 °C and 79% relative humidity for various durations (0, 12, 24, 36, 48, and 60 h). A saturated potassium bromide solution was used to maintain the desired humidity level. After the conjugation process, samples were collected and stored in a desiccator. The browning index of conjugates aqueous solution was analyzed at 420 nm by PE Lambda 35 ultraviolet spectrophotometer (PerkinElmer, Cambridge, USA). The degree of grafting of conjugates was measured by the o-phthalic aldehyde method (Sheng et al. 2020). The grafting of WPI and mannose during the Maillard reaction was analyzed using fluorescence spectroscopy and infrared (FTIR) spectroscopy.

For the TPP-WPI-Man conjugates, the carboxyl group of TPP was activated by dissolving TPP (25.74 mg, 0.06 mmol), 1-ethyl-3-(3-dimethylaminopropyl) carbodiimide (34.15 mg, 0.18 mmol), and N-hydroxysuccinimide (20.73 mg, 0.18 mmol) in 10 mL of DMSO solution and stirring for 8 h. The TPP carboxyl activation solution was added to the WPI-Man conjugates (100 mL, 10 mg/mL), which were then stirred for 24 h at room temperature. The reaction product was then lyophilized after being purified for 48 h with a 500 Da dialysis bag. After lyophilization, TPP-WPI-Man conjugates were obtained. The structure of the TPP-WPI-Man conjugates was measured using ^1^H-NMR spectroscopy, X-ray photoelectron (XPS) spectroscopy, X-ray diffraction (XRD) spectroscopy, and FTIR spectroscopy.

### Preparation of AXT-loaded nanoparticles (AXT@WPI-Man and AXT@TPP-WPI-Man)

In brief, 20 mL of deionized water was used to dissolve 200 mg of WPI-Man conjugates or TPP-WPI-Man conjugates. Separately, 4 mg of astaxanthin was thoroughly dissolved in 4 mL of a 2:1 acetone: dichloromethane organic solvent solution. The astaxanthin solution was then combined with the WPI-Man conjugate or TPP-WPI-Man conjugate solution and blended for 2 min at 10,000 r/m in an Ultra-Turrax T25 high-speed mixer (IKA, Staufen, Germany). The resulting mixture was treated with ultrasonic energy for 15 min (600 W, 5 s on/5 s off) in an ice bath to homogenize the solution. The solution was then centrifuged at 2000 r/m for two minutes after the organic solvent was eliminated using rotary evaporation at 37 °C. The AXT@WPI-Man and AXT@TPP-WPI-Man nanoparticles were then lyophilized and stored in the dark at 4 °C for further characterization and evaluation.

### Characterization of AXT@WPI-Man and AXT@TPP-WPI-Man nanoparticles

The morphology of the nanoparticles was examined using a SU8010 scanning electron microscope (Hitachi, Tokyo, Japan). The encapsulation efficiency, UV-damaged photostability, and thermal stability were assessed following our previous method with slight modifications (Chen et al. 2021; Song et al. 2021).

### Analysis of macrophages targeting and mitochondria colocalization

The hydrophobic Nile Red was used to substitute astaxanthin to prepare Nile Red@WPI-Man and Nile Red@TPP-WPI-Man nanoparticles. At a density of 1 × 10^5^ cells per well, Raw264.7 cells were seeded in 12-well microplates and cultivated for 24 h. After that, the culture medium was switched out for fresh medium containing Nile Red, Nile Red@WPI-Man, Nile Red@TPP-WPI-Man, or Nile Red@TPP-WPI-Man pretreated with mannose (100 μg/mL) for 4 h. The cells were then treated with Hoechst 33,342 (10 g/mL) for 10 min. Three PBS buffer solution washes were performed on the cells before fluorescent inverted microscope images were taken (Nikon Corp., Tokyo, Japan).

To assess the mitochondria-targeting property and colocalization of the nanoparticles, a confocal laser fluorescence microscope (Leica Microsystems GmbH., Wetzlar, Germany) was utilized. Nile Red-loaded nanoparticles were observed using a microscope with excitation at 530 nm and emission at 635 nm. The RAW 264.7 cells were treated with fresh culture medium containing Nile Red-loaded nanoparticles (20 μg/mL) and incubated for 2 and 4 h. Following incubation, the media containing the samples was taken out then the cells were given a 100 nmol/L dose of Mito-tracker green solution. After a 30-min staining period, NIH ImageJ software (Bethesda, MD, USA) was used to assess fluorescence colocalization.

### Biocompatibility and cellular antioxidant activity of AXT-loaded nanoparticles in vitro

The biocompatibility of free astaxanthin, AXT@WPI-Man, and AXT@TPP-WPI-Man was determined using an MTT assay. Different concentrations of free astaxanthin, AXT@WPI-Man, and AXT@TPP-WPI-Man solutions (2.5, 5, 10, 20, and 40 g/mL) were applied to the cells. The fresh culture media was then mixed with MTT solution (20 μL, 5 mg/mL) and incubated at 37 °C for 4 h after 24-h incubation. Subsequently, 150 μL of DMSO was added to replace the medium, and the absorbance was calculated at a wavelength of 570 nm. For further evaluation, RAW 264.7 cells were plated on 12-well plates at a density of 1 × 10^5^ cells per well and treated for 24 h with fresh culture medium containing AXT@WPI-Man and AXT@TPP-WPI-Man (20 μg/mL). The medium was removed and cells were then exposed to H_2_O_2_ (400 μmol/L) for 30 min. Next, the cells were cultured with DCFH-DA (excitation wavelength = 502 nm; emission wavelength = 530 nm) (10 μmol/L) to measure ROS at 37 °C for another 30 min. In addition, the cells were stained with JC-1 fluorescent dye (10 μmol/L) at 37 °C for 20 min to assess mitochondrial membrane potential. Fluorescence images were observed, and the intensity was quantified using ImageJ software.

Moreover, a Seahorse XF-8 extracellular flux analyzer (Seahorse Bioscience, Billerica, MA, USA) from Agilent Technologies was used to measure the oxygen consumption rate (OCR) and extracellular acidification rate (ECAR) of RAW 264.7 cells in real-time to assess the impact on mitochondrial function.

### Therapeutic outcomes in vivo against ulcerative colitis

The Dalian Polytechnic University Institutional Animal Care and Use Committee approved all animal experimentation before it was carried out. Male BALB/c mice (aged 6–7 weeks, SPF, weighing 20–22.5 g) were placed into 7 groups, each consisting of 10 mice: (1) healthy control group, (2) DSS model group, (3) astaxanthin group (astaxanthin content: 200 mg/kg), (4) WPI-Man carrier group, (5) AXT@WPI-Man nanoparticle group (astaxanthin content: 200 mg/kg), (6) TPP-WPI-Man carrier group, and (7) AXT@TPP-WPI-Man nanoparticle group (astaxanthin content: 200 mg/kg). The mice of the healthy control and DSS model groups had unrestricted access to water from days 1 to 14. The astaxanthin, carrier, and nanoparticle groups received the corresponding samples via oral gavage. From days 15 to 21, the DSS control, astaxanthin, carrier, and nanoparticle groups were orally administered 4% (w/v) DSS to cause severe colitis. From days 8 to 13, the disease activity index, which includes weight loss, stool consistency, and fecal occult blood (rated from 0 to 3), was noted.

The mice were euthanized on day 21, and samples of whole blood, colon tissue, and major organs were collected for further analysis. The collected tissue and organ samples were fixed in 4% (w/v) paraformaldehyde and stained with hematoxylin and eosin (H&E) to observe histopathological changes. A 1 cm length section of the colon located near the cecum was collected for HE staining to ensure accurate histologic analysis. The levels of major inflammatory cytokines, which include tumor necrosis factor-alpha (TNF-α), interleukin-1β (IL-1β), interleukin-6 (IL-6), and interleukin-10 (IL-10), in the serum were examined by specific enzyme-linked immunosorbent assay (ELISA) kits from Nanjing Jiancheng Bioengineering Institute (Nanjing, China). Colon tissue (0.1 g) was mixed with physiologic saline (w:v = 1:9) to prepare the tissue homogenate. After centrifuging at 2500 r/m for 10 min, the supernatant was collected to assess the protein concentration. In addition, colon tissues were examined for the presence of biochemical markers like glutathione (GSH), inducible nitric oxide synthase (iNOS), malondialdehyde (MDA), and myeloperoxidase (MPO) using biochemical kits, as directed by the manufacturer (Nanjing Jiancheng Bioengineering Institute, Nanjing, China).

### Immunofluorescence assay

Frozen slices of colon tissue, approximately 6 μm thick, were fixed with acetone for 15 min. Subsequently, they were permeated with 0.5% Triton-X-100 for 20 min and blocked with 5% goat serum for 30 min. Three PBS washes were performed after each stage. After that, the primary antibody (CD86 or CD163) was incubated with the sections for an entire night at 4 °C. The slices were then treated with secondary antibodies tagged with fluorescent dyes at 37 °C for 1 h after being washed with PBS containing Tween-20. After 5 min of DAPI (10 μg/mL) staining, the slices were exposed to a fluorescence inverted microscope (Nikon Corp., Tokyo, Japan) to obtain the necessary pictures.

### Statistical analyses

The data were presented as the mean ± standard deviation (SD) and measured in triplicate. Graphs of the sample data were generated using GraphPad Prism 8 software (GraphPad Software, San Diego, CA, USA). GraphPad Prism 8 software (GraphPad Software, San Diego, CA, USA) was used to create graphs of sample data. Statistical analysis was conducted using one-way ANOVA.

## Results and discussion

### Characterization of AXT@WPI-Man and AXT@TPP-WPI-Man nanoparticles

**Fig. 1:**
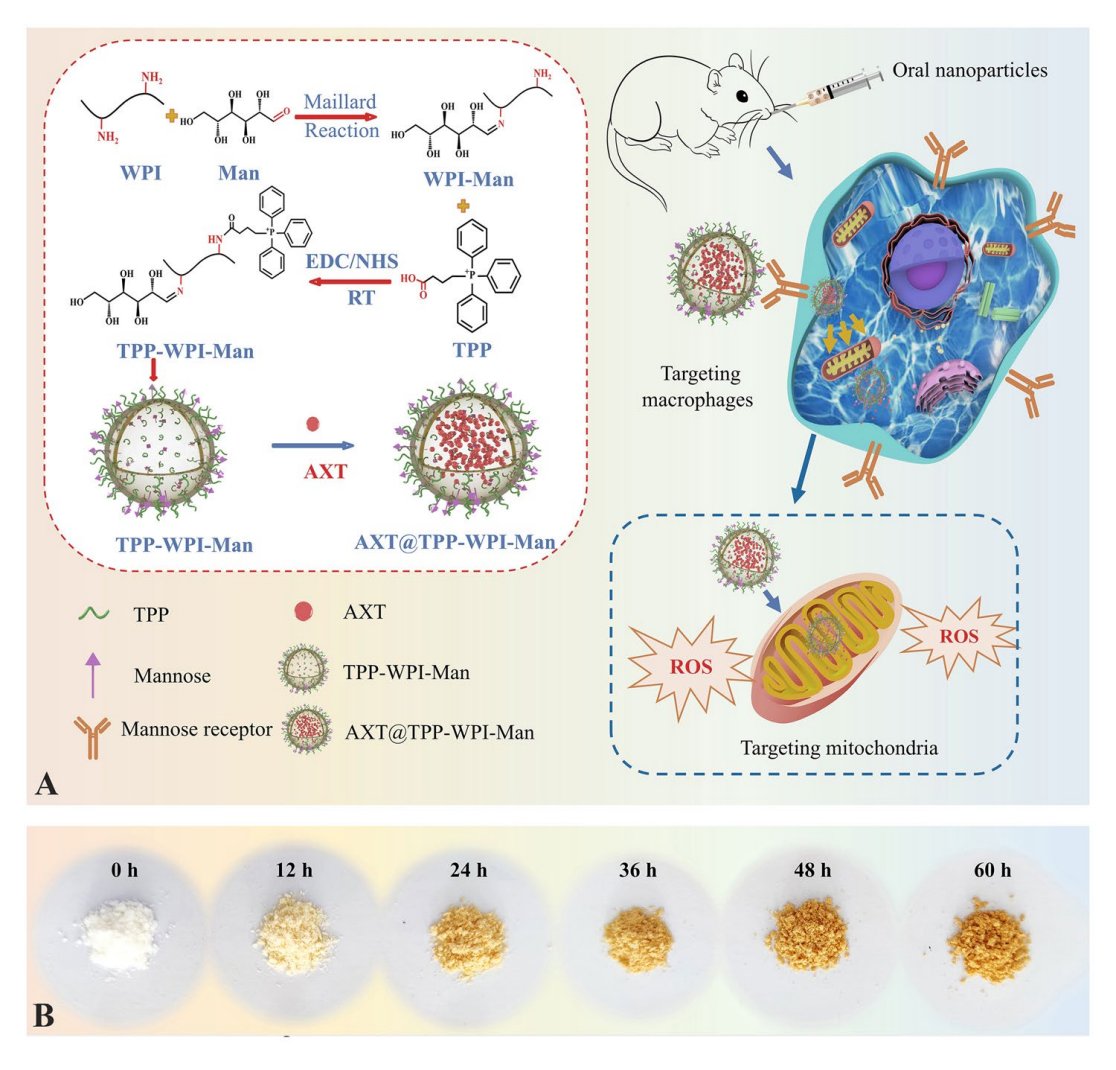
**A** Schematic diagram of the preparation of macrophage and mitochondria dual-targeting astaxanthin-loaded nanoparticles (AXT@TPP-WPI-Man) for colonic inflammation alleviation. **B** WPI-Man conjugates after Maillard reaction at 0, 12, 24, 36, 48, and 60 h

**Fig. 2:**
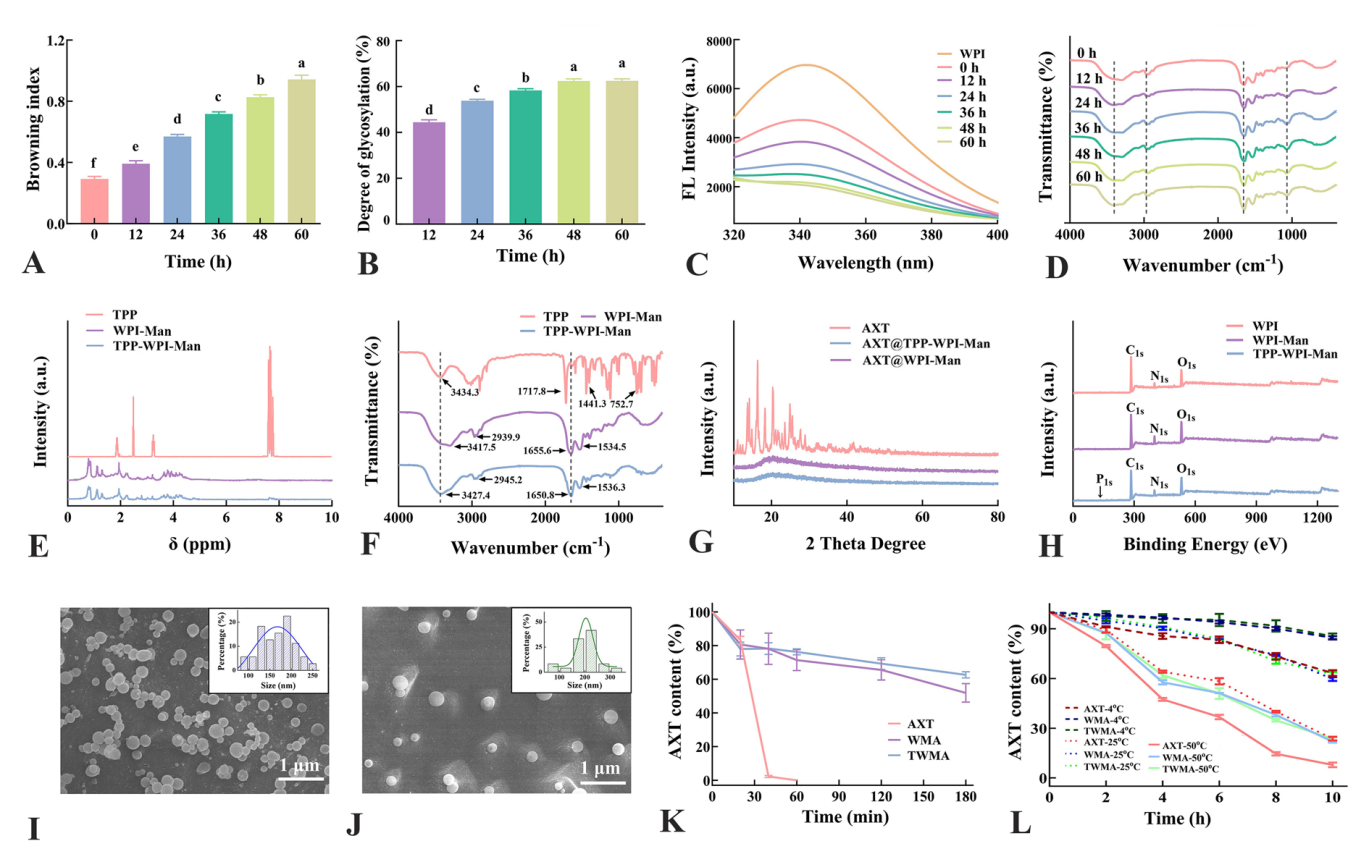
**A** Browning index, **B** degree of grafting, **C** fluorescence spectra, **D** FTIR spectra of WPI-Man conjugates with different reaction times. **E** ^1^H NMR spectra of TPP, WPI-Man, and TPP-WPI-Man. **F** FTIR spectra of TPP, WPI-Man, and TPP-WPI-Man. **G** XRD patterns of TPP, WPI-Man, and TPP-WPI-Man. **H** XPS spectra of WPI, WPI-Man, and TPP-WPI-Man. SEM images of **I** AXT@WPI-Man and **J** AXT@TPP-WPI-Man. Stability experiments of **K** UV-radiation time and **L** temperature. *n* = 3 per group. Different letters indicate significant differences at *P* < 0.05

A novel type of AXT-loaded nanoparticle designed to target both macrophages and mitochondria was synthesized using glycosylation of WPI-Man conjugates modified with TPP for colonic inflammation alleviation (Fig. 1A). The Maillard reaction, responsible for the formation of chromophores and the development of a brown color, served as an indicator of the extent and rate of the reaction (Oliveira et al. 2016). The color of the glycosylation WPI gradually darkened (Fig. 1B), while the browning index showed increased absorbance over the course of 0 to 60 h (Fig. 2A). Simultaneously, the degree of glycosylation also increased with reaction time and reached a relatively constant level (Fig. 2B). Notably, the degree of glycosylation at 48-h incubation showed no significant difference compared to that at 60 h, which could be attributed to a decrease in available amino groups for reaction or the occurrence of steric hindrance (Markman and Livney 2012). The fluorescence intensity of the WPI-Man conjugates was lower than that of WPI (Fig. 2C), suggesting the mannose molecules shielded the tryptophan residues, particularly in samples with longer reaction times (Pirestani et al. 2018). The FTIR spectra revealed a wide absorption at 3600–3200 cm^−1^ corresponding to O–H and N–H groups (Fig. 2D). The absorption peaks at 2949–2941 cm^−1^ indicated the antisymmetric stretching of C–H in the CH_2_ and CH_3_ groups. The C–O stretching vibration was associated with the sharp band seen at 1260–1000 cm^−1^, and its intensity increased with the extension of reaction time. Furthermore, the intensity of the peaks associated with the amide II band (N–H bending) and amide III bands (C–N stretching and N–H bending) at 1600–1500 cm^−1^ and 1450–1200 cm^−1^, respectively, decreased, suggesting the amine groups of the protein were consumed by covalent binding (Sheng et al. 2020). Given the degree of glycosylation in WPI, the WPI-Man conjugates incubated for 48 h were selected for subsequent experiments to ensure the preservation of the protein’s structural integrity.

The structural characterization of TPP-WPI-Man was conducted using ^1^H NMR. The peaks corresponding to the protons of TPP, specifically (CH_2_ (a)), (CH_2_ (b)), and (CH_2_ (c)), were observed in the ranges of 2.43–2.53 ppm, 1.77–1.91 ppm, and 3.16–3.32 ppm, respectively (Fig. 2E) (Han et al. 2014). Although there were slight shifts due to the introduction of TPP, the peaks from the WPI-Man conjugate spectra were still present in the TPP-WPI-Man ^1^H NMR spectrum. In the TPP-WPI-Man ^1^H NMR spectrum, signals around 7.56–7.81 ppm, attributed to the protons of the TPP phenyl rings, confirming the formation of TPP-WPI-Man (Han et al. 2014). The structures of TPP, WPI-Man, and TPP-WPI-Man were also analyzed using FTIR spectra (Fig. 2F). The phenyl ring stretching vibrations of TPP were represented by the distinctive peaks at 1718.4, 1441.3, and 752.7 cm^−1^, corresponding to C = O, C–O–H/C = C. The peaks of WPI-Man at 3417.5 and 2939.9.6 cm^−1^ represented O–H and N–H stretching vibrations, respectively.

The C = O stretching vibration peak (amide I band) and N–H stretching vibration peak (amide II band) were attributed to the peaks at 1655.6 and 1534.5 cm^−1^, respectively. Importantly, the amide I and amide II bands absorption peaks in the TPP-WPI-Man spectrum were remarkably increased compared to those in the WPI-Man spectrum, indicating successful grafting of TPP onto WPI-Man (X Han et al. 2018). The crystalline diffraction patterns of astaxanthin, AXT@WPI-Man, and AXT@TPP-WPI-Man were analyzed, revealing that astaxanthin had a crystalline structure. However, in the spectra of AXT@WPI-Man and AXT@TPP-WPI-Man, the crystalline structure disappeared, and new diffraction peaks at approximately 20° were seen, demonstrating successful embedding of astaxanthin (Fig. 2G). Furthermore, XPS analysis demonstrated that TPP-WPI-Man consisted of carbon (C), oxygen (O), nitrogen (N), and phosphorus (P), with the P originating from TPP (Fig. 2H). TPP-WPI-Man was also found to contain carbon (C), oxygen (O), nitrogen (N), and phosphorus (P), with the P coming from TPP, according to XPS analysis (Fig. 2H). Collectively, these findings supported the TPP modification of WPI-Man conjugates as successful.

The SEM images in Fig. 2I, J display the shape of AXT@WPI-Man and AXT@TPP-WPI-Man nanoparticles, respectively. Both types of nanoparticles exhibited a spherical shape, with average particle sizes of 169.4 ± 36.3 nm for AXT@WPI-Man and 206.1 ± 39.2 nm for AXT@TPP-WPI-Man. The larger size of AXT@TPP-WPI-Man nanoparticles can be attributed to the presence of modified TPP, which occupies additional spatial positions. The encapsulation efficiency values for AXT@WPI-Man and AXT@TPP-WPI-Man nanoparticles were 74.4 ± 1.5% and 77.8 ± 1.8%, respectively (Supplementary Fig. S1A), indicating no significant difference in encapsulation efficiency after TPP modification.

Zeta potential measurements for AXT@WPI-Man and AXT@TPP-WPI-Man were − 36.1 mV and − 29.8 mV, respectively, suggesting the introduction of a positive charge due to TPP modification (Supplementary Fig. S1B) (Han et al. 2018). To evaluate the potential benefits of encapsulation, the stability of AXT@WPI-Man and AXT@TPP-WPI-Man under UV radiation and temperature was investigated. As the UV irradiation time increased, the retention rates of free astaxanthin, AXT@WPI-Man, and AXT@TPP-WPI-Man dropped, as shown in Fig. 2K. After 180 min of UV exposure, the retention rates of AXT@WPI-Man and AXT@TPP-WPI-Man were 51.8% ± 4.5% and 62.6% ± 1.5%, respectively, which were significantly higher than that of free astaxanthin. In addition, Fig. 2L demonstrates that the retention of AXT@WPI-Man and AXT@TPP-WPI-Man was significantly higher than that of free astaxanthin with increasing temperature and heating time, indicating the encapsulation of astaxanthin protected it from oxidation and degradation.

### Macrophages targeting and mitochondria colocalization analysis

**Fig. 3:**
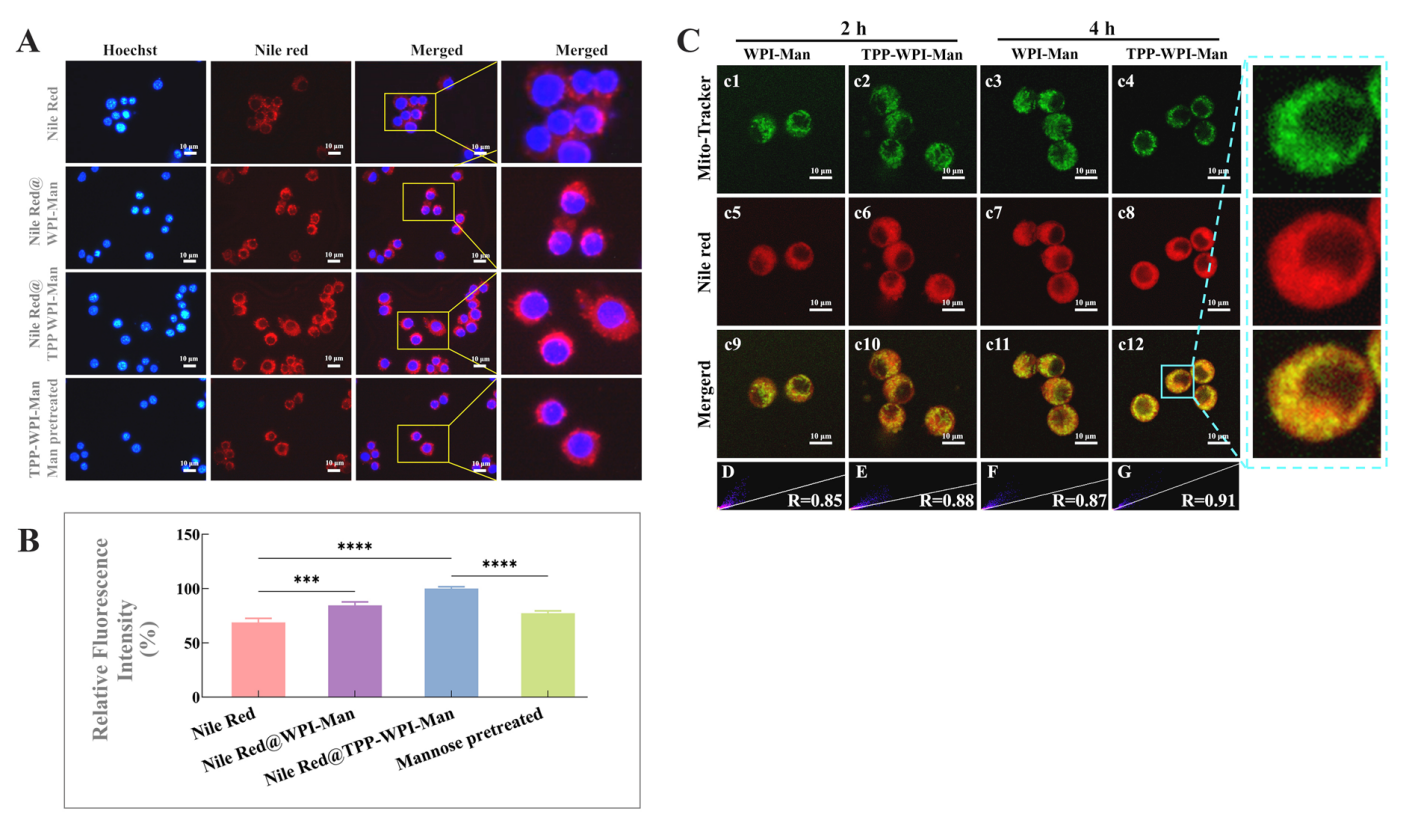
**A** Fluorescent images of cellular uptake profiles in Raw264.7 macrophages after being treated with Red@WPI-Man, Red@TPP-WPI-Man, and Red@TPP-WPI-Man pretreated with mannose. **B** Relative fluorescence intensity across different treatment groups. Mitochondrial colocalization (**C**) of WPI-Man and TPP-WPI-Man for 2 h and 4 h by pretreatment with different fluorescent dyes. (Green: Mito-Tracker Green, Red: Nile red). Pearson’s correlation coefficient (R) for the degree of mitochondrial (red) and carrier (green) colocalization in **D** WPI-Man and **E** TPP-WPI-Man at 2 h or **F** WPI-Man and **G** TPP-WPI-Man at 4 h. Scale bars = 10 μm. *n* = 3 per group (ns > 0.5, *P ≤ 0.05, **P ≤ 0.01, ***P ≤ 0.001, ****P ≤ 0.0001)

Mannose can be recognized by abundant mannose receptors on macrophages (Linehan et al. 2000). In this study, a competitive inhibition experiment was used to study the macrophage targeting effect of nanoparticles. As seen in Fig. 3A, the cells with Nile Red@WPI-Man showed only mild red fluorescence after 4 h of incubation, while noticeably increased red fluorescence in the Nile Red@TPP-WPI-Man treated cells. The cells were first primed with mannose solution for 1 h before being incubated with Nile Red@TPP-WPI-Man to further establish better macrophage colocalization ability of Nile Red@TPP-WPI-Man due to the mannose receptors. Compared with the Nile Red@TPP-WPI-Man treated groups, the red fluorescence intensity of Nile Red@TPP-WPI-Man with the pretreatment obviously decreased to 77.34% (Fig. 3B). The results strongly indicated that Nile Red@TPP-WPI-Man had a higher ability to target macrophages because it selectively targeted macrophages via mannose receptors.

To evaluate the targeting capabilities of AXT@TPP-WPI-Man nanoparticles, Mito-Tracker Green was utilized to stain mitochondria, while Nile Red was employed to label the AXT@WPI-Man and AXT@TPP-WPI-Man nanoparticles for tracing purposes. After a 2-h incubation period, Nile Red@WPI-Man nanoparticles exhibited weak orange fluorescence in the mitochondria of the cells. On the contrary, treatment with Nile Red@TPP-WPI-Man nanoparticles resulted in stronger orange fluorescence, indicating a higher colocalization capability with the mitochondria (Fig. 3c11 and c12). The intensity of the orange fluorescence from the Nile Red@WPI-Man and Nile Red@TPP-WPI-Man nanoparticles gradually increased as the incubation period was extended from 2 to 4 h. Moreover, the Pearson’s correlation coefficients (R) of Nile Red@TPP-WPI-Man nanoparticles were 0.88 at 2 h and 0.91 at 4 h, which were higher than those of Nile Red@WPI-Man nanoparticles (0.85 and 0.87) (Fig. 3D–G). This indicates that TPP-WPI-Man nanoparticles exhibited better fluorescence colocalization in the mitochondria. Hence, the results clearly demonstrated the preferential targeting and accumulation of TPP-WPI-Man nanoparticles in the mitochondria. Similar findings were reported by Zhang et al. (2022), who constructed TPP-modified cauliflower-like carriers to enhance the biocompatibility of astaxanthin and effectively alleviate colitis. These results suggest that WPI-Man nanoparticles decorated with TPP possess mitochondrial targeting capabilities.

### Determination of biocompatibility, ROS, mitochondrial membrane potential, and mitochondrial energy metabolism of AXT@TPP-WPI-Man nanoparticles in vitro

Using the MTT assay, the biocompatibility of free astaxanthin, AXT@WPI-Man, and AXT@TPP-WPI-Man was evaluated (Supplementary Fig. S2). After 24 h of incubation, the cell viability increased with the dose concentration for both AXT@WPI-Man and AXT@TPP-WPI-Man, with AXT@TPP-WPI-Man demonstrating higher cell viability compared to AXT@WPI-Man. This outcome can be attributed to the TPP modifications, which facilitated cellular internalization and localization in the mitochondria, thereby achieving targeted delivery and enhancing the bioavailability of astaxanthin.

**Fig. 4:**
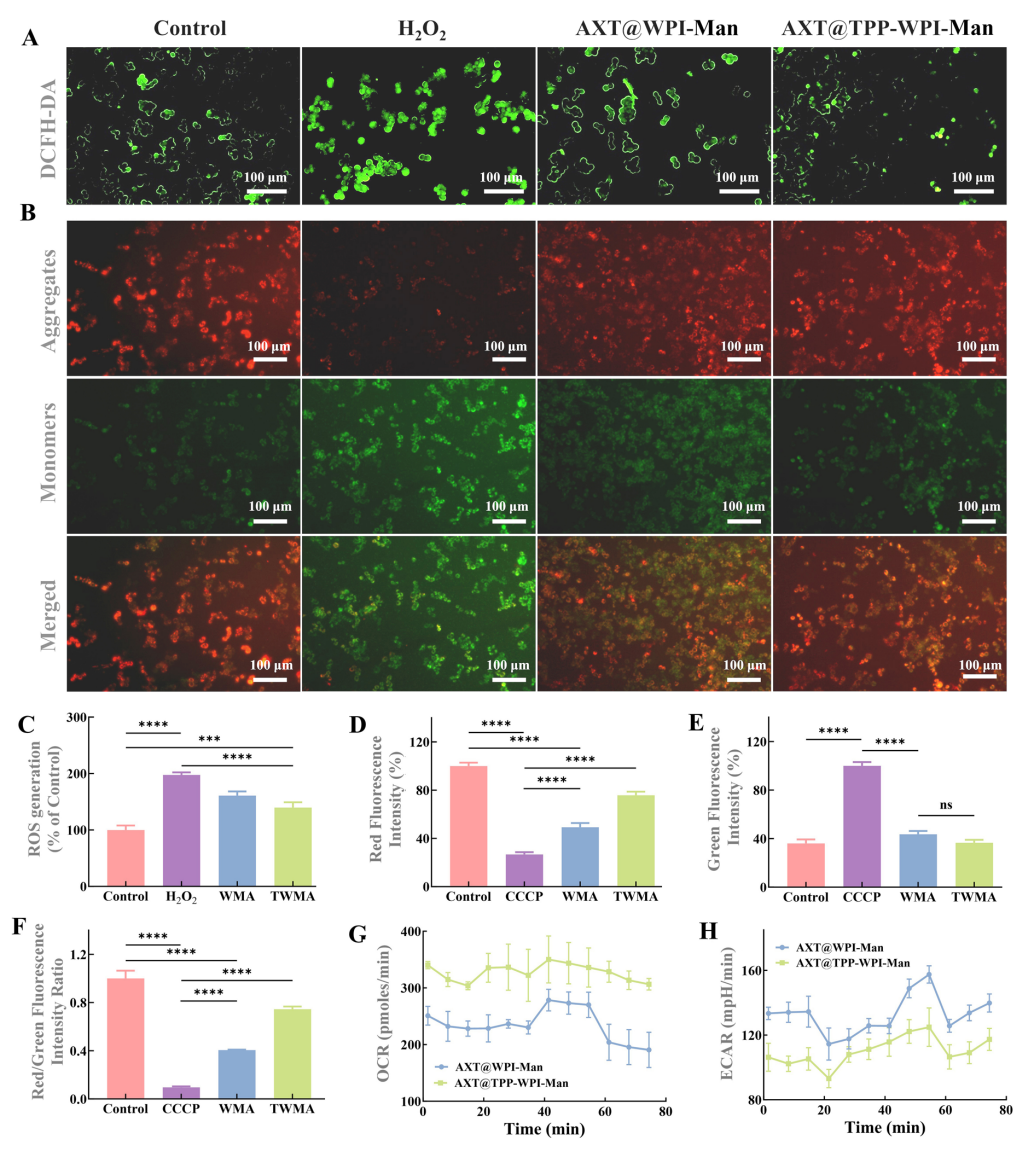
Fluorescence images of Raw 264.7 cells stained with the DCFH-DA probe after treatment with **A** DMEM (control), H_2_O_2_, AXT@WPI-Man + H_2_O_2,_ and AXT@TPP-WPI-Man + H_2_O_2_. **B** Fluorescent images of mitochondrial membrane potential for Raw 264.7 cells stained with JC-1 probe before (control) and after treatment with CCCP, AXT@WPI-Man (WMA), and AXT@TPP-WPI-Man (TWMA). **C** Relative fluorescence intensity across different treatment groups. The intensity of **D** red fluorescence and **E** green fluorescence. **F** The ratio of red/green-fluorescence intensity. Real-time OCR (**G**) and ECAR (**H**) values of Raw 264.7 cells treated with AXT@WPI-Man and AXT@TPP-WPI-Man. Scale bars = 50 μm. *n* = 3 per group (ns > 0.5, *P ≤ 0.05, **P ≤ 0.01, ***P ≤ 0.001, ****P ≤ 0.0001). Scale bar = 100 μm

Excessive production of ROS, induced by H_2_O_2_, plays a significant role as an inflammatory mediator that can lead to DNA, protein, and lipid damage and trigger inflammation (Yu et al. 2020).To explore the possible antioxidant properties of AXT@WPI-Man and AXT@TPP-WPI-Man nanoparticles against oxidative stress, ROS generation was measured using DCFH-DA probes, which generated highly fluorescent DCFH upon reacting with intracellular ROS (Shanmugapriya et al. 2019). The results depicted in Fig. 4A revealed a substantial increase in the fluorescence intensity of ROS to 197.64 ± 3.63% when cells were exposed to H_2_O_2_ compared to control cells, indicating an imbalance in redox reactions. However, treatment with AXT@WPI-Man and AXT@TPP-WPI-Man nanoparticles resulted in a decrease in ROS levels in RAW 264.7 cells to 160.91 ± 6.06% and 139.58 ± 7.75%, respectively, illustrating the nanoparticles’ capability to alleviate ROS levels induced by H_2_O_2_ (Fig. 4C). Importantly, the AXT@TPP-WPI-Man nanoparticles, which possess mitochondrial targeting properties, exhibited a more pronounced protective effect by mitigating ROS production. This finding is consistent with a study conducted by Zhang et al. demonstrating that TPP-modified AXT-loaded cauliflower-like carriers effectively alleviate intracellular ROS production, thereby suggesting the mitochondrial targeting property can increase its protective effect of astaxanthin by reducing ROS production (Zhang et al. 2022).

Mitochondria, cell organelles consisting of two-layer membranes, are crucial for aerobic respiration, which powers biochemical reactions within the cell. The change in cellular mitochondrial membrane potential was assessed by observing the fluorescent signal of the dye JC-1. Red fluorescence indicates a high potential of the mitochondrial membrane, whereas green fluorescence represents a lower potential. As shown in Fig. 4B, the ratio of red to green fluorescence significantly decreased (9.58 ± 0.68%) upon stimulation with CCCP, a mitochondrial membrane depolarizer. Remarkably, AXT@WPI-Man and AXT@TPP-WPI-Man nanoparticles therapy improved the red/green-fluorescence ratio compared to CCCP stimulation (40.52 ± 0.36% and 74.45 ± 1.81%) (Fig. 4D–F). Moreover, the red/green-fluorescence intensity ratio of AXT@TPP-WPI-Man nanoparticles exceeded that of AXT@WPI-Man nanoparticles, indicating AXT@TPP-WPI-Man nanoparticles effectively maintained the cellular mitochondrial membrane potential. Chen et al. (2021) also demonstrated that TPP-modified nanocarriers effectively maintained mitochondrial membrane potential, enhancing the protective effect of astaxanthin against H_2_O_2_-induced damage in RAW 264.7 cells.

In general, energy metabolism plays a critical role in determining fundamental cellular functions. ATP is produced via glycolysis and mitochondrial respiration, with oxidative phosphorylation (OXPHOS) in the mitochondria as the primary source. Mitochondrial aerobic respiration in RAW 264.7 cells treatment with AXT@TPP-WPI-Man was significantly higher than in cells incubated with AXT@WPI-Man (Fig. 4G), leading to improved ATP production. Consistently, cells incubated with AXT@TPP-WPI-Man exhibited lower ECAR values than cells incubated with AXT@WPI-Man (Fig. 4H). When cellular mitochondria are damaged and unable to produce ATP via OXPHOS, increased glycolytic activity is required to sustain the energy supply. The results indicate that TPP-modified nanoparticles have a greater ability to alleviate the inhibitory effect of H_2_O_2_ on mitochondrial function in RAW 264.7 cells.

### Therapeutic efficiency in the ulcerative colitis mouse model

**Fig. 5:**
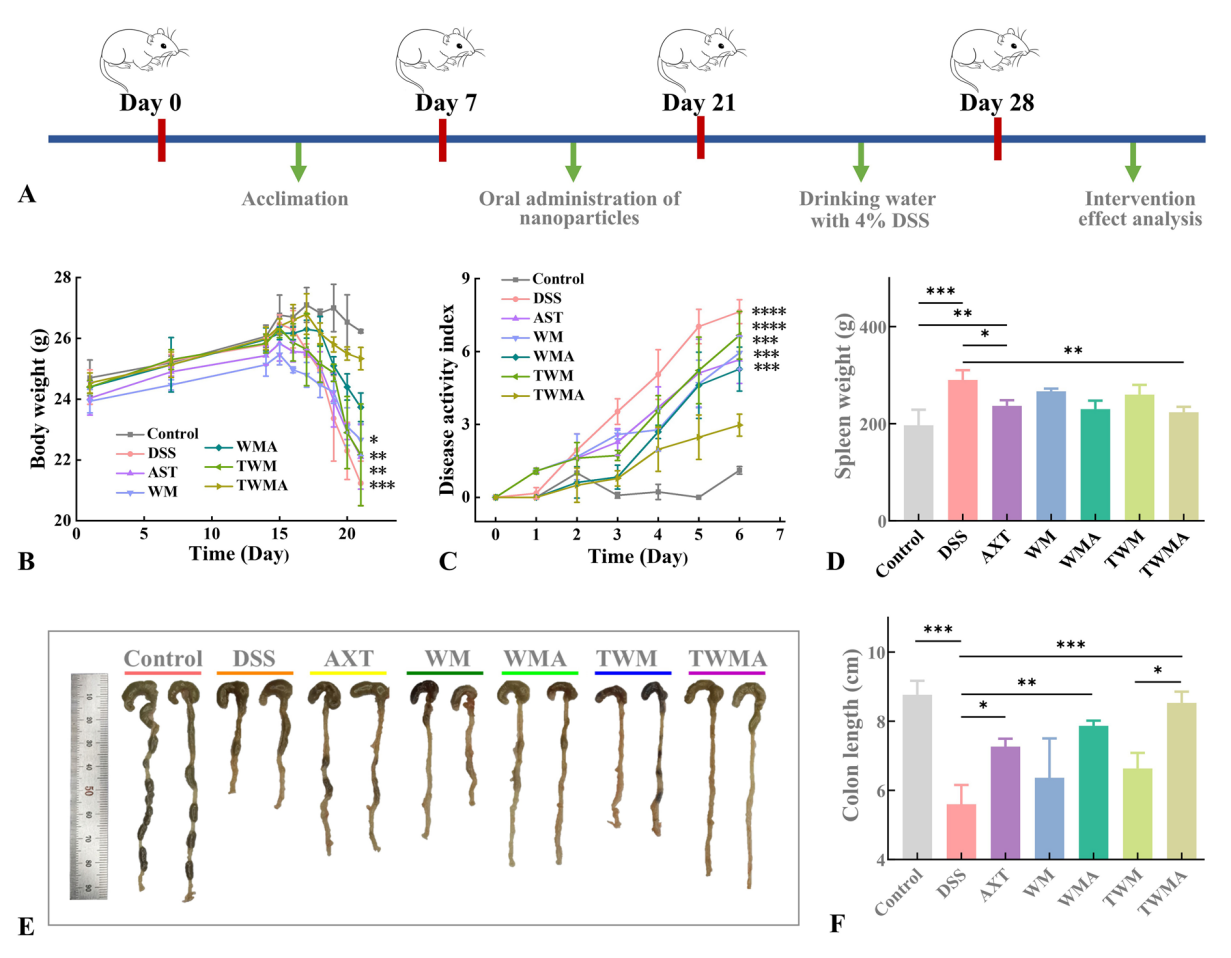
**A** Schematic illustration of colitis mice induced with 4% DSS in vivo and nutrition intervention with AXT, WPI-Man, AXT@WPI-Man, TPP-WPI-Man and AXT@TPP-WPI-Man. **B** Time-dependent variations in body weight. **C** Disease activity index scores of each group from days 8 to 14. **D** Spleen weight. **E** Photographs of colons and **F** colon length of mice in different groups. *n* = 3 per group (ns > 0.5, *P ≤ 0.05, **P ≤ 0.01, ***P ≤ 0.001, ****P ≤ 0.0001)

During the entire experimental period, except for the negative control and DSS-positive groups, the mice were orally administered free AXT, WPI-Man, AXT@WPI-Man, TPP-WPI-Man, and AXT@TPP-WPI-Man from days 8 to 21. Subsequently, a 4% (w/v) DSS solution was provided for drinking to all groups except the control from days 21 to 28 (Fig. 5A). Weight loss is a commonly used clinical indicator (Wang et al. 2013), and it was observed that body weight began to decrease on day 24 after DSS intervention. The DSS-treated mice experienced significant body weight loss, while the administration of AXT@TPP-WPI-Man nanoparticles noticeably mitigated this weight loss (Fig. 5B). The severity of inflammation was further evaluated by scoring the disease activity index, which included parameters, such as weight loss, stool consistency, and fecal occult blood (Turner et al. 2009). The disease activity index of the DSS model group was remarkably higher than that of the healthy control group, while the administration of AXT@TPP-WPI-Man nanoparticles showed an obvious decreasing trend (Fig. 5C). The spleen, an essential immunologic organ that indirectly reflects the level of inflammation was assessed by measuring spleen weight. The spleen weight of the DSS model group (290 ± 16 mg) was significantly larger than that of the healthy control group, which had the lowest spleen weight (196 ± 26 mg) (Fig. 5D). Compared to the healthy control group, the sample-treated DSS groups appeared a mild increase in spleen weight, and the AXT@TPP-WPI-Man nanoparticles exhibited the lowest spleen weight of the groups (223.2 ± 9.3 mg).

The gross observations (Fig. 5E) and length measurements (Fig. 5F) of the excised colons revealed a significant reduction in colon length in colitis mice (5.60 ± 0.45 cm) compared to a healthy colon (8.76 ± 0.32 cm). However, the administration of AXT@WPI-Man nanoparticles (7.93 ± 0.21 cm) and AXT@TPP-WPI-Man nanoparticles (8.53 ± 0.26 cm) alleviated the symptoms of colonic shortening, with AXT@TPP-WPI-Man nanoparticles demonstrating a superior effect compared to AXT@WPI-Man nanoparticles. These results indicate that nutritional intervention with AXT@WPI-Man and AXT@TPP-WPI-Man nanoparticles effectively alleviated the clinical symptoms in DSS-induced colitis mice, with AXT@TPP-WPI-Man nanoparticles showing a more pronounced effect.

**Fig. 6:**
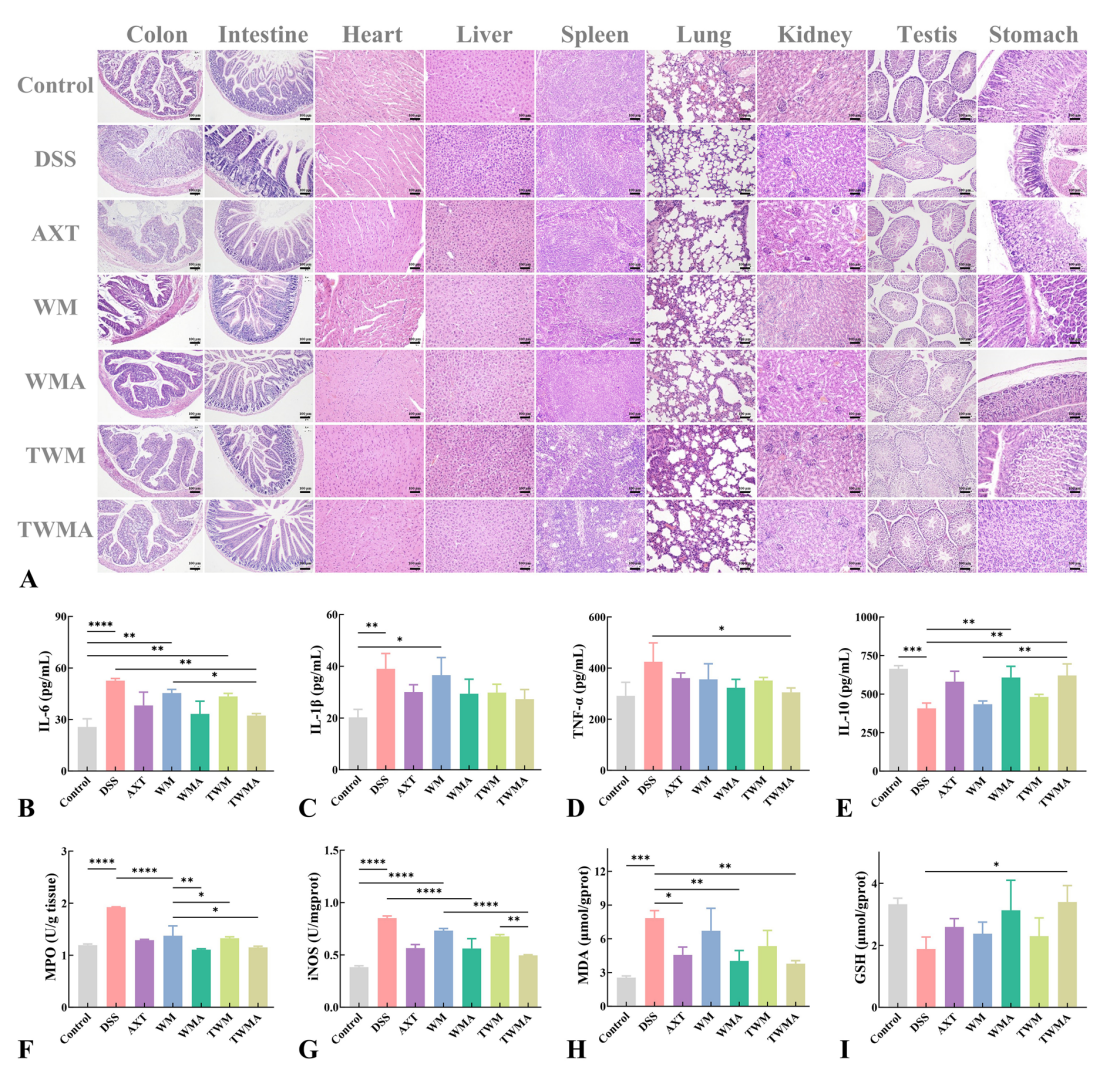
Histologic and inflammatory cytokine analyses. **A** H&E histologic sections of the main organs of mice across different groups. Scale bar = 100 μm. The expression levels of **B** IL-1β, **C** IL-6, **D** IL-10, **E** TNF-α, **F** MPO, **G** iNOS, **H** MDA, and **I** GSH across different groups. *n* = 3 per group (ns > 0.5, *P ≤ 0.05, **P ≤ 0.01, ***P ≤ 0.001, ****P ≤ 0.0001)

To evaluate the pathologic changes in major organs of colon colitis mice, we explored histologic changes by H&E staining (Fig. 6A). Histologic evaluation of the colon is mainly based on three parameters: the severity of inflammation, crypt damage, and ulceration (Laroui et al. 2014). The colonic tissue sections of the healthy control group had a normal colon tissue structure, complete intestinal crypt, compact columnar epithelium, and a clear layer between the mucosa and submucosa. In contrast to the healthy control group, the structure of the crypt in the group treated with DSS was remarkably destroyed, with structural deformation in the crypt and inflammatory cell infiltration. After treatment with other sample groups, the colonic tissues showed much lower degrees of inflammation, alleviating of crypt structure damage and weakened infiltration of inflammatory cells. Notably, almost normal histologic microstructure was observed in colon tissues from the AXT@TPP-WPI-Man nanoparticles, comparable to healthy controls. Similar results were found in tissue from the small intestine as in the colon. Furthermore, no histologic structural damage was found in the heart, lung, liver, spleen, kidney, testis, and stomach in other treatment groups, suggesting that the nanoparticles have good biocompatibility.

Increasing research indicates that macrophages play a key role in the development of ulcerative colitis by secreting many pro-inflammatory substances like IL-1β, IL-6, and TNF-α (Bauché et al. 2018; Bosma et al. 2016). Therefore, macrophages are considered one of the important target cells in the treatment of ulcerative colitis. As shown in Fig. 6B–D, the DSS model group induced significant secretion of IL-1β, IL-6, and TNF-α compared to the healthy control group, which are typical features of colonic inflammation. After treatment with other samples, the mice exhibited inhibition in the secretion of the major pro-inflammatory cytokines IL-1β, IL-6, and TNF-α, and AXT@TPP-WPI-Man nanoparticles inhibited the inflammatory factors more significantly. Moreover, the anti-inflammatory cytokine IL-10 markedly increased after treatment with AXT@TPP-WPI-Man, indicating that AXT@TPP-WPI-Man nanoparticles could efficiently reduce inflammation in ulcerative colitis mice (Fig. 6E).

MPO, primarily produced and secreted by neutrophil granulocytes, is commonly quantified for the biochemical assessment of tissue inflammation, and it has been proven to be an essential indicator for judging the severity of colitis (Schwab et al. 2014). As presented in Fig. 6F, the colonic MPO activities in the DSS model group (1.92 ± 0.01 U/g tissue) were dramatically higher than that in the healthy control group (1.19 ± 0.02 U/g tissue), while the AXT@TPP-WPI-Man nanoparticles (1.15 ± 0.02 U/g tissue) exhibited the lowest colonic MPO content among all the other groups. The production of nitric oxide (NO), induced by iNOS, is intimately related to the development of ulcerative colitis and increased NO production leads to tissue damage and inflammation (Deng et al. 2020). As illustrated in Fig. 6G, increased expression of iNOS was found in the DSS model group (0.38 ± 0.01 U/gprot) compared to the healthy group (0.85 ± 0.02 U/gprot), while the expression of iNOS in the astaxanthin nanoparticle group significantly decreased, with a better effect observed in the AXT@TPP-WPI-Man group (0.49 ± 0.01 U/gprot).

The levels of MDA and GSH in the serum of mice were determined in order to further investigate the intervention effect of nanoparticles on oxidative stress in mice with enteritis (Fig. 6H, I). The content of MDA in the DSS model group increased from 2.54 ± 0.12 to 7.85 ± 0.54 μmol/gprot compared to the healthy control group, while the level of GSH significantly decreased from 3.32 ± 0.15 to 1.88 ± 0.31 μmol/gprot. The results suggest that increased levels of oxidative stress and colonic inflammation jointly aggravate intestinal damage. As expected, intervention with AXT@TPP-WPI-Man nanoparticles markedly decreased the level of MDA (3.79 ± 0.21 μmol/gprot) and increased the level of GSH (0.49 ± 0.01 μmol/gprot).

### Macrophage polarization in DSS mice

**Fig. 7:**
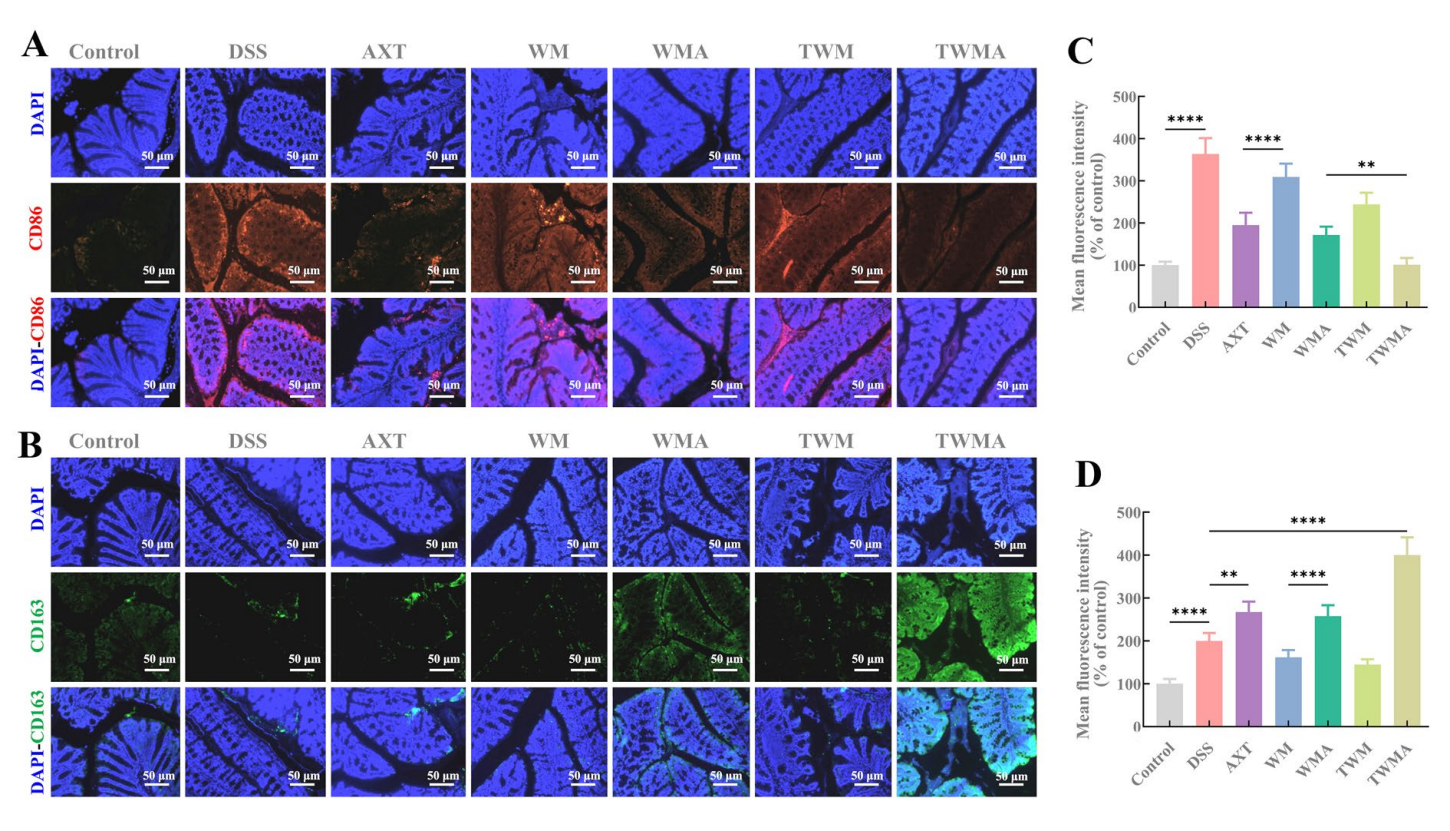
The effect of nanoparticles on macrophage polarization in DSS-mice. Sections of colon tissues were immunostained with DAPI (blue), **A** CD86 (red), and **B** CD163 (green). The mean fluorescence intensity of **C** CD86, and **D** CD163. Scale bar = 50 µm. *n* = 4 per group (ns > 0.5, *P ≤ 0.05, **P ≤ 0.01, ***P ≤ 0.001, ****P ≤ 0.0001)

The tissue microenvironment provides multiple signals that can directly influence the polarization of monocytes into either the M1 or M2 macrophage phenotype (Na et al. 2019). M2 macrophages have anti-inflammatory activity, aid in wound healing, and prevent fibrosis, in contrast to M1 macrophages, which are known for promoting inflammation and antimicrobial activity (Wynn and Barron 2010). Macrophage polarization is crucial in the development of inflammation (Zhang et al. 2020). To evaluate the subset of macrophage subpopulations, dual immunofluorescence staining of CD86 and CD163, which serve as molecular markers for M1- and M2-polarized macrophages, respectively, was performed. As seen in Fig. 7A and B, the protein expression of CD86 was significantly elevated in the colon tissue of the DSS group (363.4 ± 33.5%), while the expression of CD163 decreased (199.7 ± 16.7%) (Fig. 7C, D). However, treatment with AXT@TPP-WPI-Man nanoparticles effectively counteracted these changes induced by DSS. The protein expression of CD86 and CD163 in the AXT@TPP-WPI-Man group was 100.8 ± 14.5% and 399.8 ± 37.2%, respectively, suggesting the AXT@TPP-WPI-Man intervention alleviated colon inflammation by reducing macrophage M1 polarization and promoting M2 polarization. Similar findings were reported by Wu et al. (2021), who showed that diosgenin ameliorated colitis in mice by reducing M1 polarization and increasing M2 polarization in colonic macrophages.

## Conclusions

In conclusion, we successfully prepared and characterized novel glycosylated protein nanoparticles with macrophages- and mitochondria-targeting astaxanthin transport. The AXT@TPP-WPI-Man nanoparticles exhibited a round shape, nanometer size, negative surface charge, good dispersion, and high encapsulation efficiency. These nanoparticles facilitated the targeted enrichment of astaxanthin in mitochondria and enhanced its antioxidant activity. Furthermore, in a DSS-induced colitis mouse model, AXT@TPP-WPI-Man nanoparticles effectively alleviated inflammatory injury by modulating the polarization of macrophages. This study demonstrates the potential of AXT@TPP-WPI-Man nanoparticles as a promising delivery system for hydrophobic bioactive compounds in targeted therapy for ulcerative colitis.

## Supplementary Information

Below is the link to the electronic supplementary material.

Supplementary file1 (DOCX 94 kb)

## Data Availability

The data that support the findings of this study are available from the corresponding author on reasonable request.
