# Supplementary material for: Macrophage and mitochondrion dual-targeting astaxanthin nanoparticles prepared by Maillard reaction for colonic inflammation alleviation
Source: Mar Life Sci Technol. 2025 Feb 17;7(2):352–65. doi: 10.1007/s42995-024-00255-9 (PMC12102426; doi:10.1007/s42995-024-00255-9)
Supplement: Supplementary file 1 — Supplementary file1 (DOCX 94 kb) [file 42995_2024_255_MOESM1_ESM.docx]

**Macrophage and mitochondrion dual-targeting astaxanthin nanoparticles prepared by Maillard reaction for** **colonic inflammation alleviation**

Kangjing Liu^a,b,c,d^, Xueying Tian^a,b,c,d^, Siyuan Fei^a,b,c,d^, Yukun Song^a,b,c,d^, A. M. Abd El-Aty^e,f^, Mingqian Tan^a,b,c,d*^

*^a^**State Key Laboratory of Marine Food Processing and Safety Control, Dalian Polytechnic University, Dalian 116034 China.*

*^b^Academy of Food Interdisciplinary Science, School of Food Science and Technology, Dalian Polytechnic University, Dalian 116034 China*

*^c^National Engineering Research Center of Seafood, Dalian Polytechnic University, Dalian 116034 China*

*^d^Collaborative Innovation Center of Seafood Deep Processing, Dalian Polytechnic University, Dalian 116034, China.*

*^e^Department of Pharmacology, Faculty of Veterinary Medicine, Cairo University, 12211-Giza, Egypt*

*^f^Department of Medical Pharmacology, Medical Faculty, Ataturk University, Erzurum 25240, Turkey*

*Corresponding author: Mingqian Tan

E-mail address: [mqtan@dlpu.edu.cn](mailto:mqtan@dlpu.edu.cn)


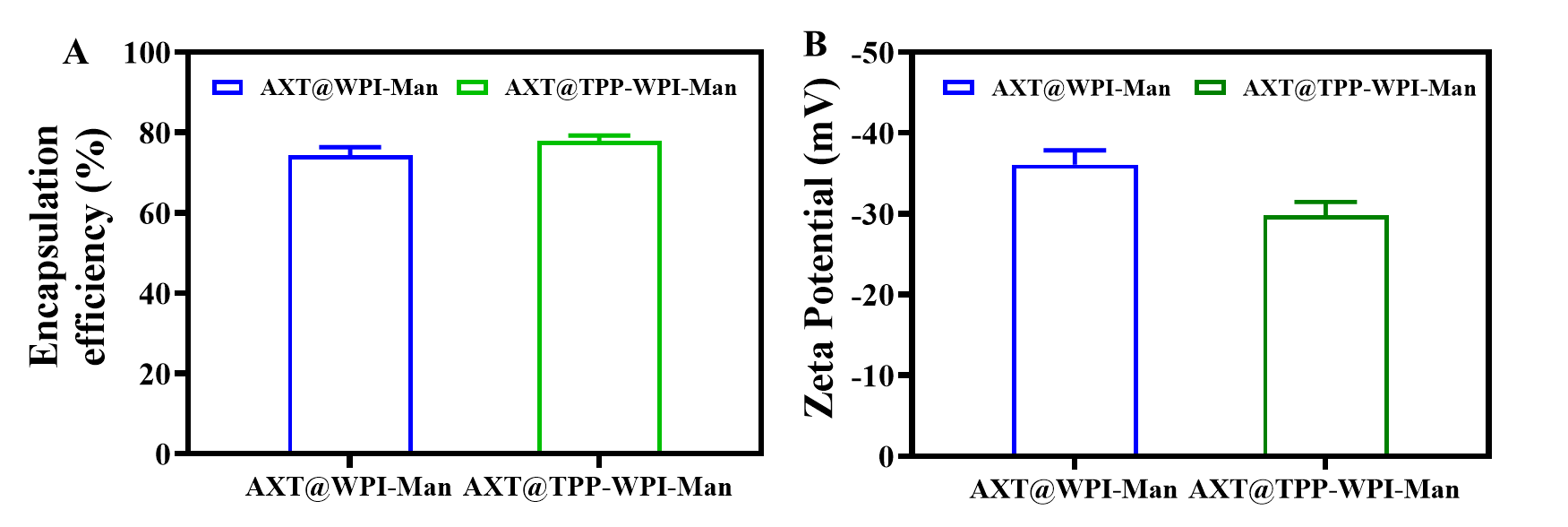


**Fig. S1 (A)** Encapsulation efficiency and (**B)** zeta potential of AXT@WPI-Man and AXT@TPP-WPI-Man.

**Fig. S2** Cell viability of Raw 264.7 cells at different concentrations of AXT, AXT@WPI-Man, and AXT@TPP-WPI-Man for 24 h. Data are represented as the mean ± SD (n=3).
